# MyD88-Dependent Signaling Contributes to Host Defense against Ehrlichial Infection

**DOI:** 10.1371/journal.pone.0011758

**Published:** 2010-07-23

**Authors:** Young-Sang Koh, Jung-Eun Koo, Amlan Biswas, Koichi S. Kobayashi

**Affiliations:** 1 Department of Cancer Immunology & AIDS, Dana-Farber Cancer Institute, Boston, Massachusetts, United States of America; 2 Department of Pathology, Harvard Medical School, Boston, Massachusetts, United States of America; 3 Department of Microbiology and Brain Korea 21 Program, Jeju National University School of Medicine, Jeju, Jeju-Do, South Korea; Charité-Universitätsmedizin Berlin, Germany

## Abstract

The ehrlichiae are small Gram-negative obligate intracellular bacteria in the family *Anaplasmataceae*. Ehrlichial infection in an accidental host may result in fatal diseases such as human monocytotropic ehrlichiosis, an emerging, tick-borne disease. Although the role of adaptive immune responses in the protection against ehrlichiosis has been well studied, the mechanism by which the innate immune system is activated is not fully understood. Using *Ehrlichia muris* as a model organism, we show here that MyD88-dependent signaling pathways play a pivotal role in the host defense against ehrlichial infection. Upon *E. muris* infection, MyD88-deficient mice had significantly impaired clearance of *E. muris*, as well as decreased inflammation, characterized by reduced splenomegaly and recruitment of macrophages and neutrophils. Furthermore, MyD88-deficient mice produced markedly lower levels of IL-12, which correlated well with an impaired Th1 immune response. *In vitro*, dendritic cells, but not macrophages, efficiently produced IL-12 upon *E. muris* infection through a MyD88-dependent mechanism. Therefore, MyD88-dependent signaling is required for controlling ehrlichial infection by playing an essential role in the immediate activation of the innate immune system and inflammatory cytokine production, as well as in the activation of the adaptive immune system at a later stage by providing for optimal Th1 immune responses.

## Introduction

The mammalian innate immune system detects the presence of microbial infection through germ line-encoded pattern recognition receptors (PRRs). Toll-like receptors (TLRs), RIG-like receptors (RLRs) and nucleotide-binding oligomerization domain-leucine rich repeat containing (NLR) proteins serve as PRRs that recognize different but overlapping microbial components, frequently referred to as pathogen-associated molecular patterns (PAMPs) [1]. TLRs detect microbial infection and activate downstream signaling cascades such as the activation of MAP kinases and NF-κB. TLR signaling and the resulting transcriptional activation of immune response genes requires the adaptor MyD88, except for TLR3 signaling, which requires another adaptor, TRIF [1]. Phylogenetic analysis groups the TLRs into six categories [2], and TLRs in each branch have similar ligands [2]; the TLR1 group, which includes TLR2/6/10, is specific for lipoproteins, TLR4 for LPS, TLR3/7 and TLR8/9 for RNA and DNA, TLR5 for flagellin, and TLR11 for protein(s) [2]. Interestingly, TLRs also localize to different cellular compartments to facilitate ligand recognition [3]. TLRs can localize to the cell surface (TLR1,2,5,6,10), endosomal surface (TLR3,7,8,9), or both (TLR4,11,12,13) [3], [4].

It has been shown that, in addition to TLRs, NLR proteins are also important for the recognition and response against bacterial infection [5], [6]. For example, Nod1 and Nod2 recognize active moieties in bacterial peptidoglycan and transcriptionally activate immune response genes via a serine/threonine kinase, Rip2 [7], [8], [9], [10]. Nod2 has been shown to respond to muramyl dipeptide (MDP), consisting of MurNAc-L-Ala-D-isoGln (or D-Glu) [11], [12]. This moiety is conserved in both Gram-positive and Gram-negative organisms, suggesting that Nod2 may confer resistance to a wide variety of bacteria. Nod1, on the other hand, has been shown to detect GM (GlcNAc-MurNAc)-tripeptide containing *meso*-DAP (diaminopimelic acid) as well as simpler peptide versions containing *meso*-DAP [7], [13]. Other NLR proteins, such as Nalp3 (Nlrp3), Nalp1 (Nlrp1) and Ipaf (Nlrc4) oligomerize upon activation to form a protein complex called the inflammasome together with the adaptor ASC (for Nalp3 and Nalp1 inflammasome) or without ASC (for Ipaf), which can activate caspase-1, resulting in efficient IL-1β processing and secretion [14], [15], [16], [17], [18], [19].

Human monocytotropic ehrlichiosis (HME) is an emerging, tick-borne disease caused by *Ehrlichia chaffeensis*, which is an NIAID Category C priority pathogen [20], [21]. *E. chaffeensis* infection in human with delayed or ineffective treatment may be fatal, particularly in immunocompromised hosts [22], [23]. The ehrlichiae are small, Gram-negative, and obligate intracellular bacteria in the family *Anaplasmataceae* and reside in endosomal compartments, primarily in macrophages or monocytes. Ehrlichiae frequently cause persistent infection in their natural hosts, which appear to utilize immune defense mechanisms against species-specific ehrlichia strains. Some examples include *E. canis* in dogs, *E. chaffeensis* in deer, *E. muris* in mice, and *E. ruminantium* in sheep, goats and cattle [20], [24]. However, ehrlichiae infection in accidental hosts may result in severe fatal disease, similar to toxic shock-like disease, as seen in in *E. chaffeensis* infection in humans or infection of the IOE (Ixodes ovatus) *Ehrlichia* strain in mice [25], [26], [27].

*E. muris*, a natural pathogen in mice, is closely related to *E. chaffeensis* and is frequently used as an *Ehrlichia* infection model in mice [28]. Infection of *E. muris* in a laboratory strain of mice such as BALB/c, AKR or C57BL/6 results in splenomegaly, anemia, and splenic follicular hyperplasia followed by the development of a low-level persistent infection [28], [29]. Both CD4 and CD8 T cells play important roles in the immune response against *Ehrlichia*, including IFN-γ production [30], [31]. Outer membrane proteins (OMPs) of the *Ehrlichia* membrane play important roles in both pathogenesis and host defense, as they are the major targets of protective antibodies and *Ehrlichia*-specific T cells [32], [33]. NKT cells are also activated during *E. muris* infection, primarily through direct recognition of microbial lipids, which takes place in a MyD88-independent manner [34].

Although *E. muris* epitopes for B and T cell activation have been characterized, neither the *E. muris* ligand(s), nor the corresponding host receptor(s), that activate the innate immune system are known [32], [33]. *E. muris* can induce inflammation in mice, but it appears that *E. muris* lacks known TLR and NLR ligands, with the exception, perhaps, of bacterial nucleic acids. Ehrlichiae lack the genes required for the biosynthesis of LPS, peptidoglycan and flagellin, which can elicit an innate immune response [35], [36] In this study, we have found that MyD88-dependent signaling is essential for protection against *E. muris* infection. Upon *E. muris* infection, dendritic cells (DCs), but not macrophages, produce large amounts of IL-12, which is important in driving the Th1 immune responses that are crucial for controlling *E. muris* infection.

## Results

### MyD88-dependent protection against *E. muris*

**Figure 1:**
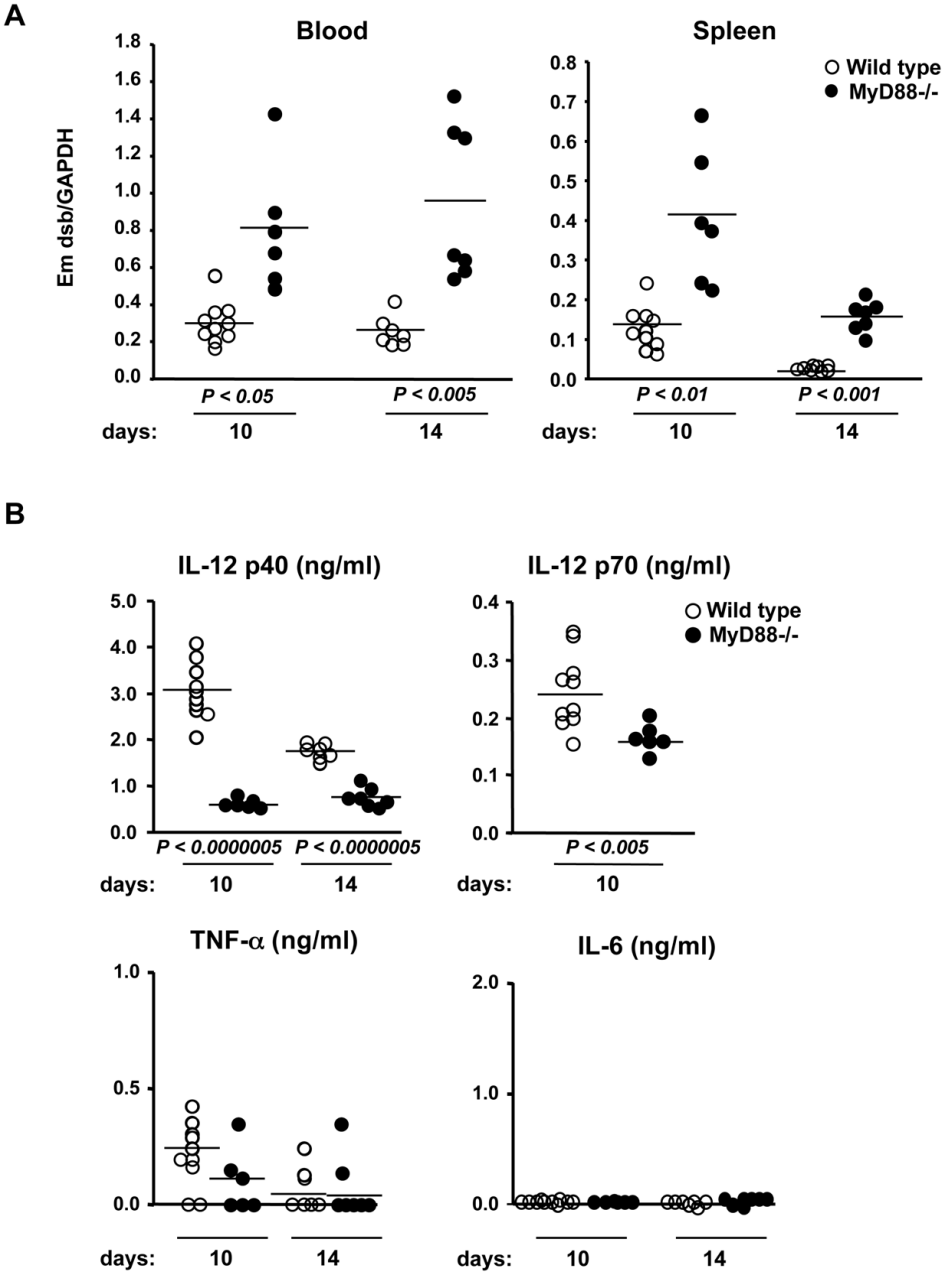
MyD88-deficient mice were susceptible to *E. muris* infection and demonstrated impaired IL-12 production *in vivo*. Age and sex-matched wild-type (*n* = 10 at day 10, *n* = 7 at day 14) and MyD88-deficient mice (*n* = 6 at day 10, *n* = 7 at day 14) were infected with *E. muris i.p.* for 10 and 14 days. (A) *E. muris* loads in blood and spleen were quantified by qPCR using primers specific for the *E. muris dsb* gene. Data were normalized by qPCR data for the GAPDH gene in host genomic DNA. (B) Serum IL-12 p40, IL-12 p70, IL-6, and TNF-α levels from infected mice were assessed by ELISA. A single circle represents an individual animal, and lines indicate the mean values. The *P*-values were determined by Student's *t*-test (Wild type vs. MyD88-/-). Data are representative of three independent experiments in A and B.

Although ehrlichiae infect and cause inflammation in humans, mice, and many other mammals, its recognition mechanism remains unknown. We used MyD88-deficient mice to investigate whether TLRs play a role in the detection of and protection against *E. muris*. We infected MyD88-deficient mice with *E. muris* by *i.p.* inoculation and assessed bacterial load in blood and tissues by quantitative real-time PCR (qPCR) using primers specific for the *E. muris dsb* gene. In a preliminary study, we found that the *E. muris* load did not significantly increase by day 6, but increased and peaked on day 10. Therefore, we designated the first 10 days post infection as the acute phase, and the following days up to day 14 as the convalescent phase of infection, which corresponds with previous reports [29], [37]. MyD88-deficient mice demonstrated a higher *E. muris* bacterial load in the blood in both phases (Figure 1A). In wild-type mice, *E. muris* was cleared gradually from the spleen during the convalescent phase. In contrast, MyD88-deficient mice had significantly increased bacterial loads at both day 10 and 14 post infection (Figure 1A). We measured the cytokine production in the serum of infected mice. Strikingly, there was a high level of IL-12 p40 and IL-12 p70 in the serum of wild-type mice infected with *E. muris*, whereas MyD88-deficient mice produced a significantly lower level of these cytokines (Figure 1B). We did not observe statistically significant differences in TNF-α and IL-6 levels in the serum of wild-type and MyD88-deficient mice (Figure 1B).

**Figure 2:**
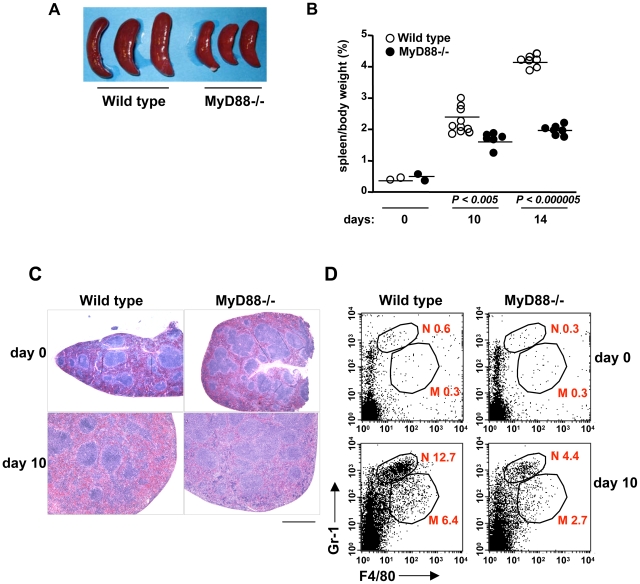
MyD88-deficient mice exhibited milder splenomegaly and leukocyte infiltration upon *E. muris* infection. Wild-type (*n* = 10 at day 10, *n* = 7 at day 14) and MyD88-deficient mice (*n* = 6 at day 10, *n* = 7 at day 14) were infected with *E. muris i.p.* for 10 and 14 days. (A and B) MyD88-deficient mice demonstrated reduced splenomegaly. Spleens were removed from the mice 10 days after *E. muris* infection and photographed in A. (B) Spleen weight was measured and normalized to body weight. A single circle represents an individual animal, and lines indicate the mean values. *P*-values were determined by Student's *t*-test (Wild type vs. MyD88-/-). (C) Spleens from infected mice were H&E-stained and photographed. Scale bar, 1 mm. (D) Reduced recruitment of neutrophils and macrophages in *E. muris*-infected MyD88-deficient mice. Surface expression of Gr-1 or F4/80 from total splenocytes was analyzed by flow cytometry. Numbers beside outlined areas indicate the percentage of Gr-1^+^ or F4/80^+^ cells among total splenocytes. N, neutrophils; M, macrophages. Data are representative of at least three independent experiments in A–D.

*E. muris* infection in wild-type mice caused severe splenomegaly that progressively became apparent after infection (Figure 2A and B). MyD88-deficient mice, however, showed a milder degree of splenomegaly (Figure 2A and B). Macroscopically, lymphoid follicles in wild-type mice were smaller and markedly irregular in shape compared to MyD88-deficient mice after infection. In contrast, lymphoid follicles in MyD88-deficient mice were larger, more uniform and distinct in shape. Additionally, the red pulp of the spleen significantly expanded in wild-type mice but only to a mild degree in MyD88-deficient mice (Figure 2C). Microscopically, this expansion was accompanied by an increased infiltration of neutrophils and macrophages into the marginal zone and the red pulp of the spleen with numerous apoptotic cells (Figure S1). Larger numbers of apoptotic cells and lymphoblasts were observed in the lymphoid follicles in wild-type mice than in MyD88-deficient mice (Figure S1). These overall histopathological findings in wild-type mice subjected to *E. muris* infection were consistent with a previous report [29]. In order to quantify the increased number of neutrophils and macrophages, we analyzed Gr-1^+^ neutrophils and F4/80^+^ macrophages by flow cytometry. The number of neutrophils and macrophages increased significantly in wild-type mice 10 days after *E. muris* infection, whereas the number of those cells increased only mildly in MyD88-deficient mice (Figure 2D). In summary, these results indicate that MyD88-dependent signaling plays an important role in the protection against *E. muris* infection by inducing inflammatory responses accompanied by IL-12 production.

### Reduced Th1 response in MyD88-deficient mice during *E. muris* infection

**Figure 3:**
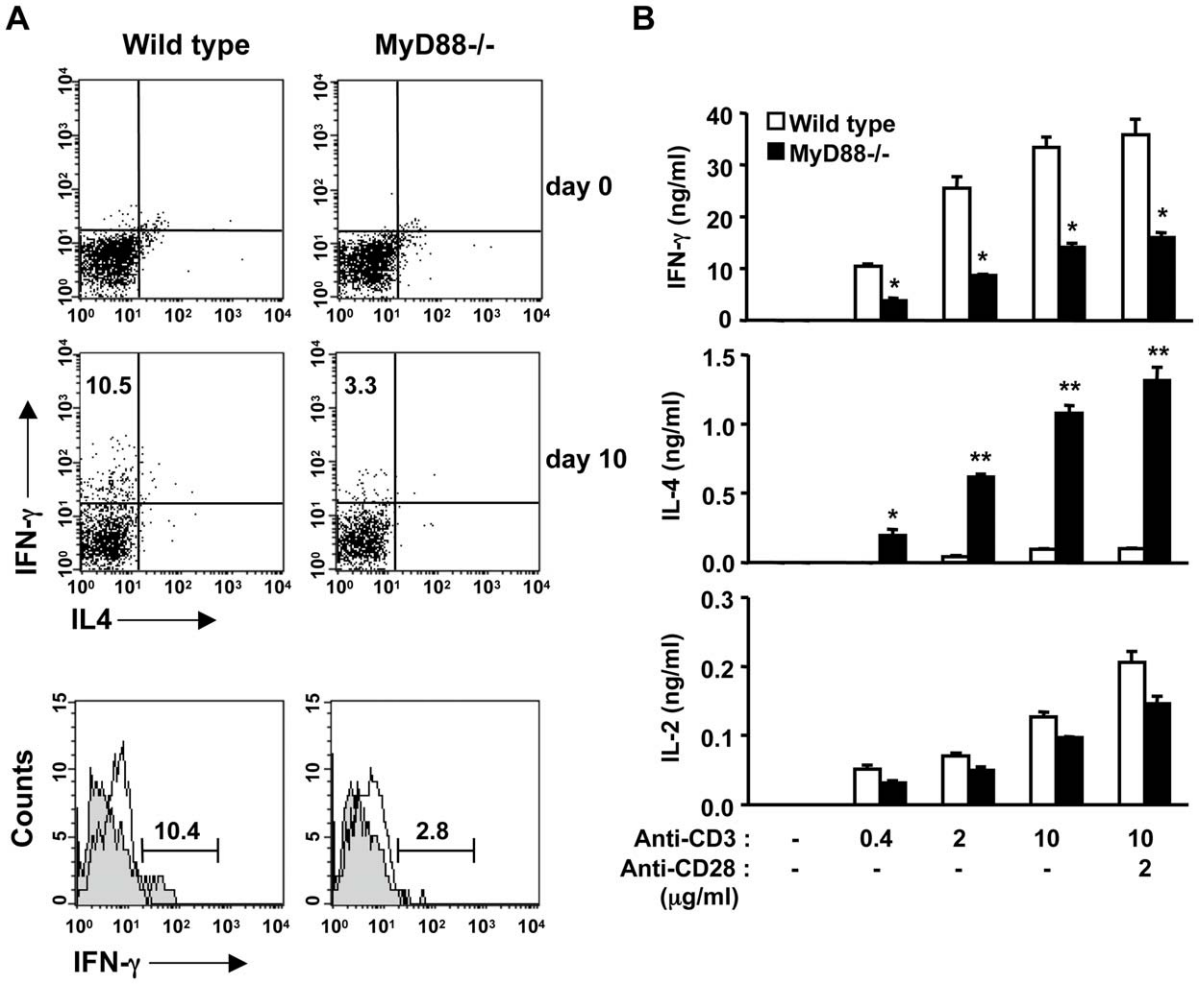
MyD88-deficient mice had reduced Th1 immune responses during *E. muris* infection. (A) The number of IFN-γ producing CD4^+^ T cells decreased in infected MyD88-deficient mice. Mice were infected with *E. muris* for 10 days. Total splenocytes were gated for CD4 surface expression, and the expression of intracellular IFN-γ and IL-4 was analyzed by flow cytometry (upper panel). Flow cytometric analysis of intracellular IFN-γ expression in splenic CD4^+^ T cells from uninfected (open histograms) or infected (shaded histograms) mice (lower panel). Numbers in top left quadrants indicate the percentage of IFN-γ producing cells in the gated CD4^+^ T cells. (B) Production of IFN-γ, IL-4, and IL-2 from splenic CD4^+^ T cells was analyzed by ELISA after restimulation with plate-bound anti-CD3 and anti-CD28 antibodies at the indicated concentrations. Error bars represent SD of triplicate samples. The *P*-values were determined by Student's *t*-test (Wild type vs. MyD88-/-). *P<0.05. **P<0.01. Data are representative of at least three independent experiments in A and B.

Since IL-12 is an important cytokine in the development of effector Th1 cells from helper CD4 T cells, reduced production of IL-12 in the serum of MyD88-deficient mice suggests that they may have an insufficient Th1 response [38]. Earlier reports have shown that a Th1 immune response is required for efficient ehrlichia clearance in mice [31], [39]. Therefore, we isolated splenocytes before and 10 days after infection of mice with *E. muris,* and IFN-γ production in gated splenic CD4 T cells was analyzed by flow cytometry. There was no significant change in the ratio of CD4 to CD8 splenic T cells during *E. muris* infection between wild-type and MyD88-deficient mice (Figure S2). However, while the percentage of IFN-γ producing CD4 T cells increased 10 days after infection in wild-type mice, the increase of IFN-γ producing cells was considerably less in MyD88-deficient mice (Figure 3A). In order to assess Th1 differentiation *ex vivo*, we purified CD4 T cells from spleens 10 days after infection and restimulated them with plate-bound antibodies against CD3 or a combination of CD3 and CD28 at different concentrations. As shown in Figure 3B, the production of IFN-γ was prominent in CD4 T cells from wild-type mice while CD4 T cells from MyD88-deficient mice produced significantly less IFN-γ. This cannot be attributed to insufficient IL-2 production in MyD88-deficient T cells, as wild-type and MyD88-deficient CD4 T cells produced similar levels of IL-2 after TCR ligation (Figure 3B), suggesting that MyD88-deficient T cells retain the intrinsic ability to produce cytokines. Indeed, MyD88-deficient CD4 T cells produced more IL-4 than wild-type CD4 T cells upon TCR ligation (Figure 3B). Taken together, these data suggest that MyD88 is required for Th1 responses during *E. muris* infection.

### DCs but not macrophages produce IL-12 efficiently upon *E. muris* infection

In order to find the major source of IL-12 production during *E. muris* infection, we generated bone marrow-derived DCs and macrophages from C57BL/6 mice and challenged them with *E. muris in vitro*. *E. muris* infection caused the generation of bacteria-containing vacuoles (morulaes) in both DCs and macrophages as observed by Diff-Quick and Hoechst33342 staining (Figure S3A). Furthermore, *E. muris* proliferated efficiently in both DCs and macrophages, as quantified by qPCR (Figure S3B). Interestingly, while DCs could produce IL-12 p40 efficiently upon *E. muris* infection, macrophages could produce only a small amount of IL-12 p40 (Figure S3C). Both cell types produced only small amounts of TNF-α and IL-6 upon *E. muris* infection (Figure S3C). Collectively, these data suggest that DCs, but not macrophages, can serve as a major source of IL-12 production during *E. muris* infection.

### The Nod-Rip2 pathway does not play a major role in the recognition of *E. muris* in DCs

Since ehrlichiae are obligate intracellular bacteria, we tested whether members of the cytoplasmic NLR protein family have the ability to detect *E. muris*. Caspase recruitment domain (CARD) containing NLR proteins (NLRC proteins) such as Nod1 and Nod2 require the kinase Rip2 for downstream signaling, which involves NF-κB activation and the induction of proinflammatory cytokines [10], [40]. Therefore, in order to reveal the role of Nod1 and Nod2 in the detection of *E. muris*, we generated bone marrow-derived DCs from Rip2-deficient mice. Cells were infected with *E. muris*, and cytokine production and bacterial loads were assessed. Levels of proinflammatory cytokines such as IL-12 p40, TNF-α, and IL-6 in wild-type DCs were comparable to that in Rip2-deficient DCs (Figure S4A). *E. muris* infection induced similar numbers of bacteria-containing vacuoles in wild-type and Rip2-deficient DCs (Figure S4B). There was also no significant difference in *E. muris* bacterial load between wild-type and Rip2-deficient DCs (Figure S4C). Collectively, these data indicate no or little involvement of Rip2 in *E. muris* detection, suggesting that the NLR family proteins Nod1 and Nod2 do not play major roles in the recognition of *E. muris* in DCs.

NLR proteins such as Nalp3 (Nlrp3) or Ipaf (Nlrc4) activate caspase-1, resulting in cleavage and maturation of IL-1β [14], [15], [16], [17], [18]. Therefore, we sought to determine whether *E. muris* infection can induce IL-1β secretion in bone marrow-derived macrophages from wild-type, Ipaf-deficient, Nalp3-deficient and Asc-deficient mice. These cells were infected with *E. muris in vitro* and IL-1β production was assessed by ELISA. We could not detect IL-1β production in macrophages from any strain of mice, suggesting that caspase-1 activating NLR proteins may not play a major role in the recognition of *E. muris* (Figure S5).

### MyD88 is required for efficient production of IL-12 from DCs upon *E. muris* infection

**Figure 4:**
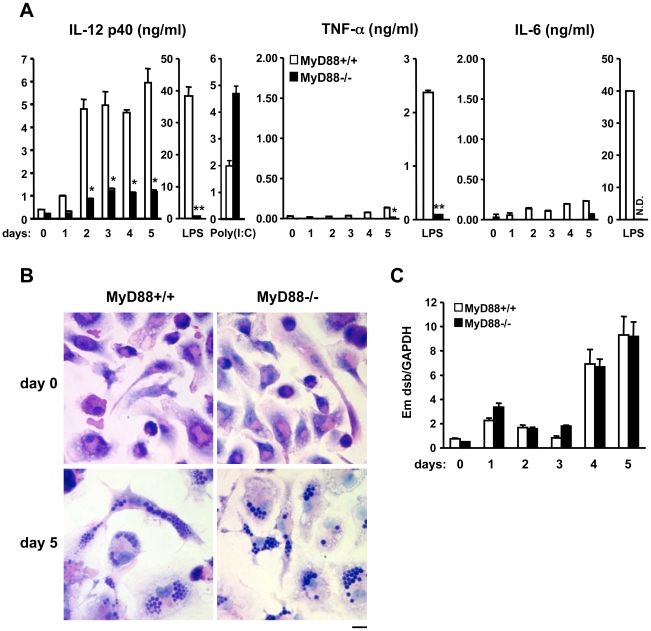
MyD88 is required for IL-12 production in *E. muris-*infected DCs. Bone marrow-derived DCs from wild-type and MyD88-deficient mice were infected with *E. muris* for the indicated time period. (A) Cytokine production from infected cells was assessed by ELISA. LPS (10 ng/ml) and poly(I:C) (50 µg/ml) stimulations were used as controls. (B) Infected cells (5 days post infection) were stained by Diff-Quick staining. Scale bar, 10 µm. (C) Bacterial loads in infected cells were quantified by qPCR using primers specific for the *E. muris dsb* gene. Data were normalized by qPCR data for the GAPDH gene in host genomic DNA. Error bars represent the SD of triplicate samples. The *P*-values were determined by Student's *t*-test (MyD88+/+ vs. MyD88-/-). *P<0.05. **P<0.01. Data are representative of at least three independent experiments in A–C. N.D., not detected.

Since DCs but not macrophages produce IL-12 p40 efficiently, we analyzed IL-12 p40 production in MyD88-deficient dendritic cells. Both wild-type and MyD88-deficient DCs were infected with *E. muris* and cytokine production was assessed by ELISA. As shown in Figure 4A, wild-type DCs produced a significantly greater amount of IL-12 p40 than MyD88-deficient DCs upon *E. muris* infection. Both TNF-α and IL-6 production decreased in MyD88-deficient DCs as compared to wild-type cells, although even wild-type DCs produced relatively low levels of these proinflammatory cytokines (Figure 4A). The decreased production of IL-12 p40 in MyD88-deficient DCs was not attributable to the difference in number of bacteria in the cells since the formation of bacterial vacuoles and the bacterial load in wild-type and MyD88-deficient DCs were comparable (Figure 4B and C).

Although less able than DCs, macrophages can produce measurable amounts of IL-12 p40 during *E. muris* infection (Figure S3C). Therefore, we challenged wild-type and MyD88-deficient macrophages with *E. muris* and assessed the production of IL-12 p40. The production of IL-12 p40 was dependent on MyD88 as MyD88-deficient macrophages failed to produce IL-12 p40 (Figure S6A). Wild-type macrophages could also produce TNF-α and IL-6 while MyD88-deficient macrophages produced these cytokines at a significantly lower level (Figure S6A). Again, this decrease was not due to failure of *E. muris* growth in MyD88-deficient macrophages since we observed similar numbers of bacteria-containing vacuoles and bacterial loads in wild-type and MyD88-deficient macrophages (Figure S6B and C).

### TLR-dependent recognition of *E. muris*

**Figure 5:**
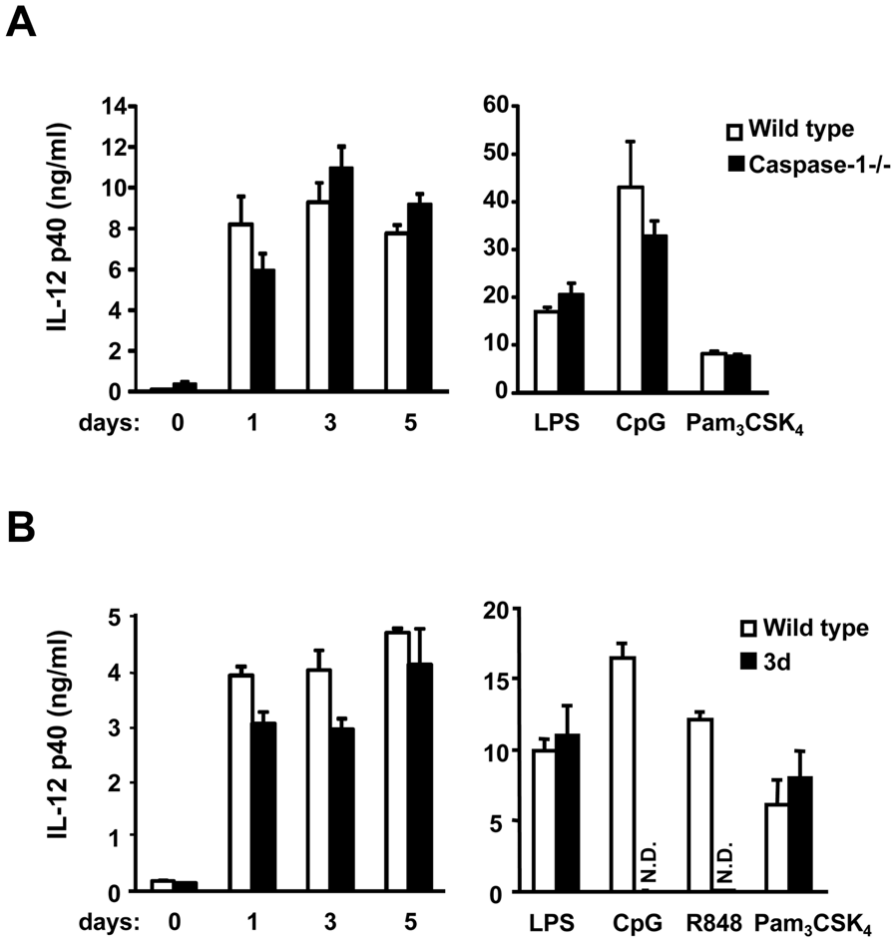
Recognition of *E. muris* in DCs is IL-1/IL-18 signaling and UNC93B1-independent. Bone marrow-derived DCs from wild-type, caspase-1-deficient (A), or 3d (B) mice were infected with *E. muris* for the indicated time period, and IL-12 p40 production from infected cells was assessed by ELISA. LPS (10 ng/ml), CpG (1 µM), R-848 (1 µM) and Pam_3_CSK_4_ (1 µg/ml) stimulations were used as controls. Error bars represent the SD of triplicate samples. Data are representative of three independent experiments in A and B. N.D., not detected.

MyD88 is required for not only TLR, but also IL-1 and IL-18 receptor signaling [41]. Caspase-1 is required for the cleavage and maturation of IL-1 and IL-18 into their biologically active forms [42], [43], [44]. In order to define the role of TLRs in the recognition of *E. muris*, we used caspase-1-deficient cells as a control against IL-1/IL-18 function. Caspase-1-deficient DCs infected with *E. muris* as well as stimulated with TLR ligands produced levels of IL-12 p40 comparable to those of wild-type DCs (Figure 5A), indicating that the IL-1/IL-18 family does not play a role in *E. muris* recognition. It has been reported that endosomal TLRs such as TLR3, 7, 8 and 9 require UNC93B1 for their endolysosomal translocation from the endoplasmic reticulum [45], [46]. Since *E. muris* grows in vacuoles in the cytoplasm, we tested whether *E. muris* recognition is dependent on UNC93B1, using triple defective (3d) mice in which UNC93B1 is mutated and non-functional, resulting in defective TLR3, 7 and 9 signaling [45]. We found that 3d DCs produced similar levels of IL-12 p40 as compared to wild-type DCs, although 3d DCs did not respond to ligands recognized by TLR9 (CpG DNA) or TLR7 (R-848) (Figure 5B). Taken together, these data indicate that *E. muris* recognition by DCs is in a caspase-1-independent and MyD88-dependent manner, suggesting that UNC93B1-independent TLR(s) may play a major role in *E. muris* recognition.

### Known TLRs do not appear to play a role in the recognition of *E. muris* in DCs

To identify which TLR is involved in the recognition of *E. muris*, we generated DCs from Tlr2, 3, 4, 5, 7, 9 and 11-deficient mice. Those cells were infected with *E. muris* and analyzed for IL-12 p40 production. Surprisingly, DCs deficient for any TLR showed a normal response to *E. muris* infection (Figure S7A and B). It has been shown that TLR1 and TLR6 cooperates with TLR2 for ligand recognition[47]. Therefore, these data indicate that TLR1, 2, 3, 4, 5, 6, 7, 9 and 11 may not play a major role in the recognition and host defense against *E. muris* infection.

## Discussion

Although adaptive immune responses during ehrlichiosis have been well characterized, the mechanism by which the bacteria activate an innate immune response has remained unexplored. Ehrlichiae are small obligate intracellular bacteria and have naturally evolved to hide from the immune system in order to survive in host cells. Intracellular bacteria grow in either in the cytoplasm or in specialized organelles that do not fuse with lysosomes, and must have strategies to escape from innate immune surveillance. Such mechanisms include the deletion or conversion of ligands into undetectable forms, rendering the organisms “invisible” from the innate immune system of host cells. For example, *Coxiella burnetii* evades TLR4 by expressing a nonstimulatory lipid A and also lacks flagellin, a ligand for TLR5 and Ipaf [48], [49]. Similarly, ehrlichiae do not possess LPS, flagellin and peptidoglycan, but are still able to induce inflammation, indicating that the mammalian innate immune system can sense ehrlichiae. In this study, we showed that MyD88 is required for host protection against *E. muris* infection. MyD88-deficient mice suffered from increased loads of *E. muris* in both blood and the spleen, which was accompanied by a decreased inflammatory response in the spleen, as well as reduced IL-12 production (Figure 1 and 2). Splenomegaly and recruitment of macrophages/neutrophils were less prominent in MyD88 deficient mice, suggesting that impaired inflammatory responses in MyD88 deficient mice correlate with less efficient bacterial clearance (Figure 2). MyD88 is also important for the induction of a proper Th1 response during *E. muris* infection, as shown in both *in vivo* and *ex vivo* assays wherein CD4 T cells from MyD88-deficient mice produced less IFN-γ (Figure 3). Therefore, since it is likely that a Th1 response, in addition to the recruitment of myeloid cells, is important for host protection against ehrlichial infection, our data suggests that MyD88-mediated recognition of ehrlichiae is important for optimal induction of Th1 immune responses.

IL-12 is an important cytokine that can induce the differentiation of Th1 effector cells from activated CD4 T cells [38]. Our data suggest that during *E. muris* infection, DCs may be a major source of IL-12. Bone marrow-derived DCs produce a large amount of IL-12 p40 in a MyD88-dependent manner, although bone marrow-derived macrophages produced only a small amount of IL-12 p40 in *in vitro* ehrlichial infection (Figure 4 and Figure S3). It has been reported recently that antigen presentation by DCs plays an important role in the immune response against ehrlichial infection [50]. Our findings indicate that in addition to ehrlichial antigen presentation, DCs may also contribute to a strong host defense against ehrlichia infection by driving strong Th1 immune responses.

Our data suggest that TLR(s) play a role in the recognition of *E. muris* since MyD88, but not caspase-1, is required for a response against *E. muris* infection in DCs (Figure 4 and 5). Recently it was reported that caspase-1 cleaves Mal/Tirap to produce its functional form. TLR2 and TLR4 responses in DCs may not require caspase-1 because a non-cleaved Mal/Tirap may still be functional in DCs, as suggested previously [51]. The identity of the TLR responsible for the recognition of ehrlichiae remains a pressing and intriguing question. Excluding bacterial DNA/RNA, ehrlichiae do not possess known TLR ligands. However, Tlr3, Tlr7 and Tlr9-deficient dendritic cells respond normally to *E. muris* infection. 3d DCs which cannot respond to ligands for TLR3, 7, 8, and 9, also produced a normal response to *E. muris* infection, suggesting that *E. muris* is not detected by any of these endosomal TLRs (Figure 5B and Figure S7B). Our data also showed that DCs from Tlr2, 4, 5 or 11-deficient mice responded to *E. muris* infection with levels of IL-12 p40 production comparable to DCs from wild-type mice (Figure S7). As for the remaining known TLRs, it has been shown that TLR1 and TLR6 heterodimerize with TLR2 to facilitate ligand recognition, and there is no murine homolog of TLR10 [47]. These indicate that the eleven TLRs that have been previously characterized may not be involved in *E. muris* recognition. It is possible that multiple TLRs contribute partially, and the sum of those effects is equivalent to the loss of functionality observed in MyD88-deficient cells. An alternate possibility is that an uncharacterized TLR accounts for the recognition of *E. muris*. Unlike humans, mice and other lower vertebrates have the TLR11 subfamily, which includes TLR12 and TLR13, whose ligands and functions are unidentified [2]. The TLR11 gene exists in humans but is not functional due to the presence of a stop codon in the leucine rich repeats [52]. It will be interesting to test if these uncharacterized TLRs are responsible for *E. muris* recognition in the future.

In addition to TLRs, there have been numerous reports of detection of intracellular bacteria by NLR proteins. For example, *Listeria monocytogenesis* infection activates Nalp3 and Nod2, *Legionella pneumophillae* infection activates Nod2, Birc1e and Ipaf, and *Salmonella typhi* infection activates Nalp3 and Ipaf [9], [17], [18], [53], [54], [55], [56], [57], [58], [59]. Interestingly, our data indicate that *E. muris* seems to be unable to activate known NLRs. *E. muris* infection in macrophages did not induce IL-1β secretion, indicating that caspase-1 activating NLR proteins such as Nalp3 or Ipaf are not involved in *E. muris* recognition (Figure S5). Also, *E. muris* infection in Rip2-deficient DCs resulted in IL-12 p40 production comparable with wild-type cells, indicating that Nod1 and Nod2 do not play major roles in *E. muris* recognition (Figure S4). Indeed, *E. muris* possess neither peptidoglycan, which contain the active moieties that are ligands for Nod1 and Nod2, nor flagellin, a ligand for Ipaf.

In conclusion, this study has demonstrated the importance of MyD88-mediated inflammatory and Th1 responses to the host defense against ehrlichial infection. Furthermore, we have identified DCs as a major source of IL-12 during *E. muris* infection. Although the specific TLR that recognizes *E. muris* remains unknown, our data suggests that obligate intracellular bacteria can elicit strong innate immune responses via TLR signaling. To our knowledge, this is the first report describing the role of the MyD88 signaling pathway in the recognition of intracellular bacteria belonging to the genus *Ehrlichia*.

## Materials and Methods

### Reagents

LPS from *Salmonella minnesota* was from Alexis. Phosphorothioate-modified CpG oligo DNA was synthesized by Integrated DNA Technology. Poly(I:C) was from Amersham Pharmacia Biotech. Flagellin from *S. typhimurium* and Pam_3_CSK_4_ were from InvivoGen. R-848 was from GLSynthesis Inc.

### Mice

MyD88, Tlr2, Tlr3, Tlr4, Tlr5, Tlr7, Tlr9, Tlr11, Rip2, caspase-1, ASC, Ipaf and Nalp3 deficient mice were established as described and kindly provided by Drs. Shizuo Akira (Osaka University), Richard Flavell, Yasunori Ogura (both at Yale University), Sankar Ghosh (Colombia University), Vishva Dixit, Sanjeev Mariathasan (both at Genentech), Douglas Golenbock (University of Massachusetts), Ann Marshak-Rothstein (Boston University) [10], [15], [17], [42], [52], [60], [61], [62], [63], [64], [65], [66]. 3d mice were established as previously (MMRRC: 010466-UCD) [45]. C57BL/6 mice were from Taconic Farm, Inc. Mice were maintained under specific pathogen-free conditions and *in vivo* infection studies were performed in microisolator cages within the ABSL-2 animal rooms in the Dana-Farber Cancer Institute Animal Research Facility. All mice were maintained and used in accordance with institutional and National Institutes of Health guidelines. All animal work was approved by the IACUC committee (#04-044 and 04-045). Harvard Medical School and Dana-Farber Cancer Institute are accredited by the American Association for the Accreditation of Laboratory Animal Care.

### Bacterial stocks and *in vitro Ehrlichia* infection of cells

*E. muris*, monocytotropic ehrlichial strain (ATCC VR-1411) was used in this study. *E. muris* was cultivated in DH82 cells with 5% FBS-supplemented DMEM at 37°C. Ehrlichiae were harvested when 90–100% of the cells were infected, and stored −80°C in sucrose-phosphate-glutamate buffer (0.218 M sucrose, 3.8 mM KH_2_PO_4_, 7.2 mM K_2_HPO_4_, and 4.9 mM monosodium glutamic acid, pH 7.0), and used for *in vitro* infection. The cells were cultured without antibiotics and *E. muris* bacterial stock was added to the cells. Infected cells were stained by Diff-Quick (Imeb Inc) or Hoechst 33342 (Invitrogen) staining. In order to produce virulent infectious stocks for *in vivo* infections, live mice were used as described previously [29], [39]. Briefly, C57BL/6 mice were inoculated *i.p.* with 0.5 ml of a 10^−1^ dilution of the frozen stock. On day 9 after inoculation, the mice were sacrificed, the spleens were harvested, and the homogenate was suspended in sucrose-phosphate-glutamate buffer. Large particles of debris were removed by centrifugation at 500 *g* for 5 min, and the supernatant was then aliquoted and stored at −80°C as a 10^−1^ stock of *E. muris*.

### Bone marrow-derived macrophages

Bone marrow-derived macrophages were prepared as described [9]. Briefly, bone marrow from tibia and femur was obtained by flushing with DMEM (Invitrogen). The medium was DMEM supplemented with 10% heat-inactivated FBS, glutamine (both from Invitrogen) and 30% L929 cell supernatant containing M-CSF. Bone marrow cells were cultured in 10 ml at an initial density of 1×10^6^ cells/ml in 100 mm petri dish (Becton Dickinson) at 37°C in humidified 10% CO_2_ for 6 days. Cells were harvested with cold PBS (Invitrogen), washed, resuspended in DMEM supplemented with 10% FBS and used at a density of 2×10^5^ cells/ml for experiments unless mentioned otherwise.

### Bone marrow-derived DCs

DCs were grown from wild-type and various knockout mice as described previously [67]. Briefly, bone marrow from tibia and femur was obtained as described above, and bone marrow cells were cultured in RPMI 1640 medium containing 10% heat-inactivated FBS, 50 µM of 2-ME, and 2 mM of glutamine supplemented with 3% J558L hybridoma cell culture supernatant containing GM-CSF. The culture medium containing GM-CSF was replaced every other day. At day 6 of culture, nonadherent cells and loosely adherent DC aggregates were harvested, washed, resuspended in RPMI 1640 supplemented with 5% FBS and used at a density of 2×10^5^ cells/ml for experiments unless mentioned otherwise.

### *Ehrlichia* challenge of mice *in vivo*

*In vivo E. muris* infection method was established previously using *E. muris* stock prepared from the spleen of the infected mice [29], [39]. Briefly, age (6–8 weeks old) and sex matched groups of mice were infected *i.p*. with *E. muris* with 250 µl of a 10^−1^ dilution of the *E. muris* stock. On the indicated days of infection, mice were sacrificed, and immune responses were assessed. Selected organs were harvested for histology, and bacterial loads were determined by qPCR. Samples of spleen were fixed with 10% formalin and stained by hematoxylin and eosin (H&E) for histopathological examination.

### CD4 T cells

CD4 T cells were isolated from spleen using a L3T4 CD4 microbeads (Mitenyi Biotec.) and stimulated with anti-CD3 and/or anti-CD28 (eBioscience) antibody for 24 hours. Culture supernatant was harvested for the measurement of IFN-γ, IL-4, and IL-2.

### Determination of Ehrlichia bacterial load in tissues and cells

Since *E. muris* is an obligatory intracellular bacterium that does not grow outside of the cells, quantification method for the *Ehrlichia* load was established previously using qPCR [29], [39]. Briefly, the ehrlichial load was determined by qPCR (with SYBR Green) of the *Ehrlichia dsb* gene (GenBank accession number:AY236484), which encodes a thio-disulfide oxidoreductase or disulphide bond formation protein of *E. muris*. Primer sequences are as follow: *E. muris* forward, TTCTTTGACTATTCCTGTGGCTAC; *E. muris* reverse, AATGACGCCTCACCGAGTAT; mouse GAPDH forward, CAACTACATGGTCTACATGTTC; and GAPDH reverse, CTCGCTCCTGGAAGATG. The substrate for amplification was DNA purified from samples using the DNeasy Tissue kit (Qiagen, Valencia, CA). qPCR was performed using the 7300 real time PCR system from Applied Biosystems. Results were normalized to GAPDH levels in the same sample and expressed as relative *Ehrlichia dsb* amounts per GAPDH amount.

### Cytokine measurement

Concentrations of murine TNF-α (R&D Systems), IL-12 p40 (BD PharMingen), IL-6 (BD PharMingen), IFN-γ (eBioscience), IL-4 (eBioscience), and IL-2 (eBioscience) in the culture supernatants or in the mouse serum were determined by ELISA according to the manufacture's instructions.

### Flow cytometric analysis

All antibodies used in this study were: NK1.1, IFN-γ, IL-4, CD4, CD8, CD80, CD86, CD11b, CD11c, CD69, CD3, B220, F4/80, Gr-1. Intracellular staining was performed after staining for surface markers. Cells were then fixed and permeabilized using permeabilization/fixation buffer (eBioscience) for 30 min at 4°C, and then stained with IFN-γ or IL-4 antibodies diluted in permeabilization buffer (eBioscience). For analysis of cell surface molecule expression, stimulated cells were washed with ice cold PBS, fixed, and stained with FITC-, PE- and APC-labeled antibodies in the presence of anti-CD16 antibody for 30 min on ice. Stained cells were washed, resuspended in PBS/1% FBS/0.05% NaN_3_, and analyzed by FACSCalibur followed by analysis using CELLQuest software (Becton Dickinson). All antibodies were obtained from eBioscience.

### Statistical analysis

All experiments were performed at least three times. All data are expressed as Mean ± SD. Data were subjected to Student's *t* test for analysis of statistical significance, and a *P* value of <0.05 was considered to be significant.

## Supporting Information

Figure S1 Histopathology of infected MyD88-deficient mice. Wild-type and MyD88-deficient mice were infected with E. muris by i.p. for 10 days. Infected spleens were H&E-stained and photographed. Scale bar, 0.1 mm. Data are representative of at least three independent experiments.
(8.27 MB TIF)

Figure S2 Similar CD4/CD8 T cell ratio between wild-type and MyD88-deficient mice during E. muris infection. Wild-type and MyD88-deficient mice were infected with E. muris for 10 days. Total splenocytes were analyzed by flow cytometry for surface expression of CD4 and CD8. Numbers in quadrants indicate the percentages of CD8+ and CD4+ cells among total splenocytes. Data are representative of at least three independent experiments.
(0.45 MB TIF)

Figure S3 DCs but not macrophages produce IL-12 p40 efficiently during E. muris infection. Bone marrow-derived DCs and macrophages were infected with E. muris for the indicated time period. (A) Infected cells (5 days postinfection) were stained by Diff-Quick and Hoechst 33342 staining. Scale bar, 10 µm. (B) Bacterial loads in infected cells were quantified by qPCR using primers specific for the E. muris dsb gene. Data were normalized by qPCR data for the GAPDH gene in host genomic DNA. (C) Cytokine production from infected cells was assessed by ELISA. Error bars represent SD of triplicate samples. LPS (10 ng/ml) stimulation was used as a control. Data are representative of three independent experiments in A-C.
(8.11 MB TIF)

Figure S4 Rip2-independent E. muris recognition in DCs. Bone marrow-derived DCs from wild-type and Rip2-deficient mice were infected with E. muris for the indicated time period. (A) Cytokine production from infected cells was assessed by ELISA. LPS (10 ng/ml) stimulation was used as a control. (B) Infected cells (5 days post infection) were stained by Diff-Quick staining. Scale bar, 10 µm. (C) Bacterial loads in infected cells were quantified by qPCR using primers specific for the E. muris dsb gene. Data were normalized by qPCR data for the GAPDH gene in host genomic DNA. Error bars represent SD of triplicate samples. Data are representative of three independent experiments in A-C.
(8.29 MB TIF)

Figure S5 IL-1β production by Asc-, Nalp3-, or Ipaf-deficient macrophages infected with E. muris. Bone marrow-derived macrophages from both wild-type and Asc-, Nalp3- or Ipaf-deficient mice were treated with LPS (10 ng/ml) for 16 hours and then infected with E. muris in vitro for the indicated time period or stimulated with 2.5 mM ATP for 3 hours. IL-1β secretion in the culture supernatants was assessed by ELISA. Error bars represent SD of triplicate samples. Data are representative of three independent experiments.
(0.18 MB TIF)

Figure S6 MyD88-dependent E. muris recognition in macrophages. Bone marrow-derived macrophages from wild-type and MyD88-deficient mice were infected with E. muris in vitro for the indicated time period. (A) Cytokine production from infected cells was assessed by ELISA. LPS (10 ng/ml) stimulation was used as a control. (B) Infected cells (5 days postinfection) were stained by Diff-Quick staining. Scale bar, 10 µm. (C) Bacterial loads in infected cells were quantified by qPCR using primers specific for the E. muris dsb gene. Data were normalized by qPCR data for the GAPDH gene in host genomic DNA. Error bars represent SD of triplicate samples. The p-values were determined by Student's t-test (MyD88+/+ vs. MyD88-/-). *P<0.05. **P<0.01. Data are representative of at least three independent experiments in A-C. N.D., not detected.
(9.43 MB TIF)

Figure S7 TLR2/3/4/5/7/9/11 do not play major roles in E. muris recognition. Bone marrow-derived DCs from wild-type, TLR2/4/5/11-deficient (A), or TLR3/7/9-deficient (B) mice were infected with E. muris for the indicated time period, and IL-12 p40 production from infected cells was assessed by ELISA. LPS (10 ng/ml), CpG (1 µM), R-848 (1 µM) and Pam3CSK4 (1 µg/ml) stimulations were used as controls. Error bars represent SD of triplicate samples. Data are representative of three independent experiments in A and B. N.D., not detected.
(1.00 MB TIF)
