# Supplementary figures and images for: MyD88-Dependent Signaling Contributes to Host Defense against Ehrlichial Infection
Source: PLoS One. 2010 Jul 23;5(7):e11758. doi: 10.1371/journal.pone.0011758 (PMC2909256; doi:10.1371/journal.pone.0011758)

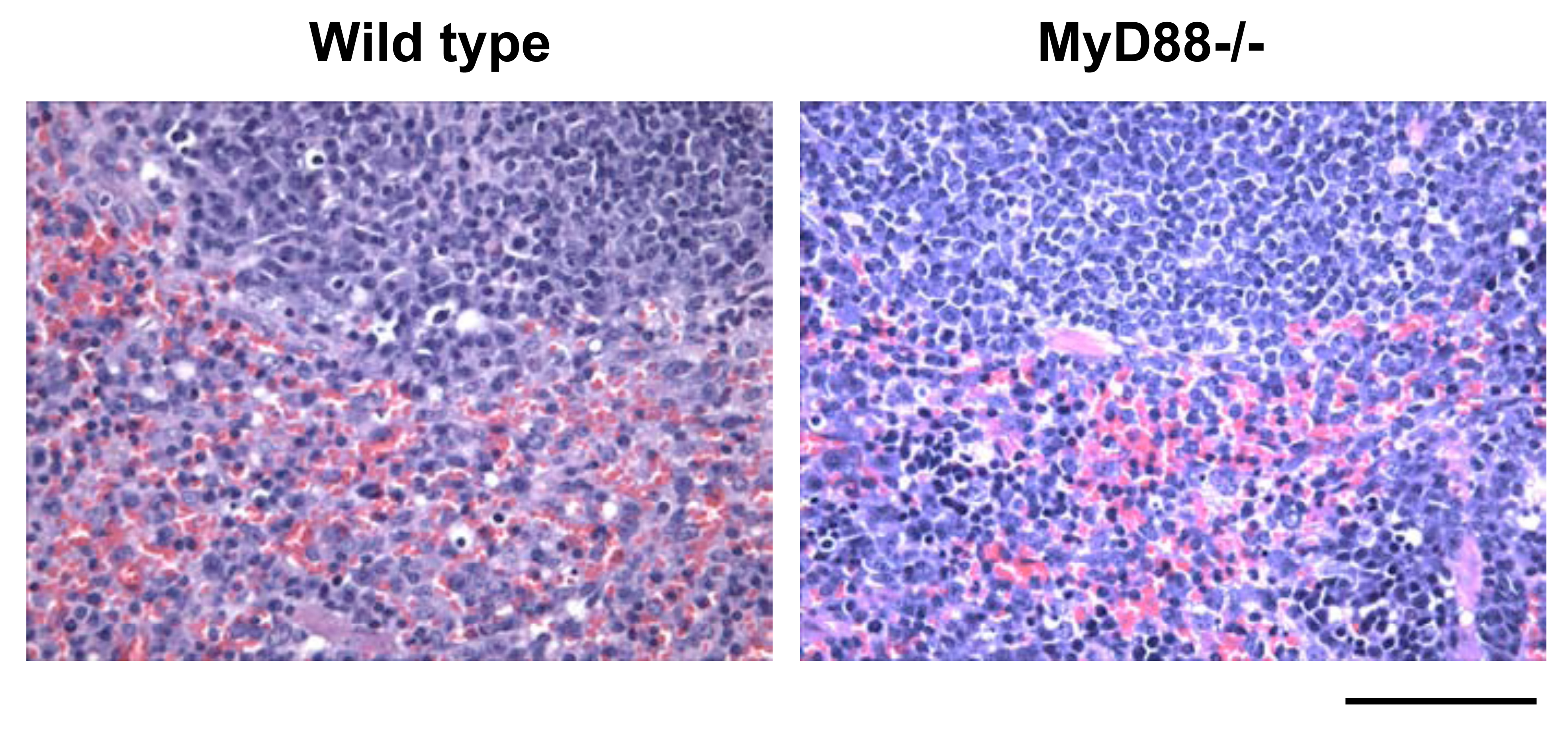

Supplement: Figure S1 — Histopathology of infected MyD88-deficient mice. Wild-type and MyD88-deficient mice were infected with E. muris by i.p. for 10 days. Infected spleens were H&E-stained and photographed. Scale bar, 0.1 mm. Data are representative of at least three independent experiments. (8.27 MB TIF) [file pone.0011758.s001.tif]

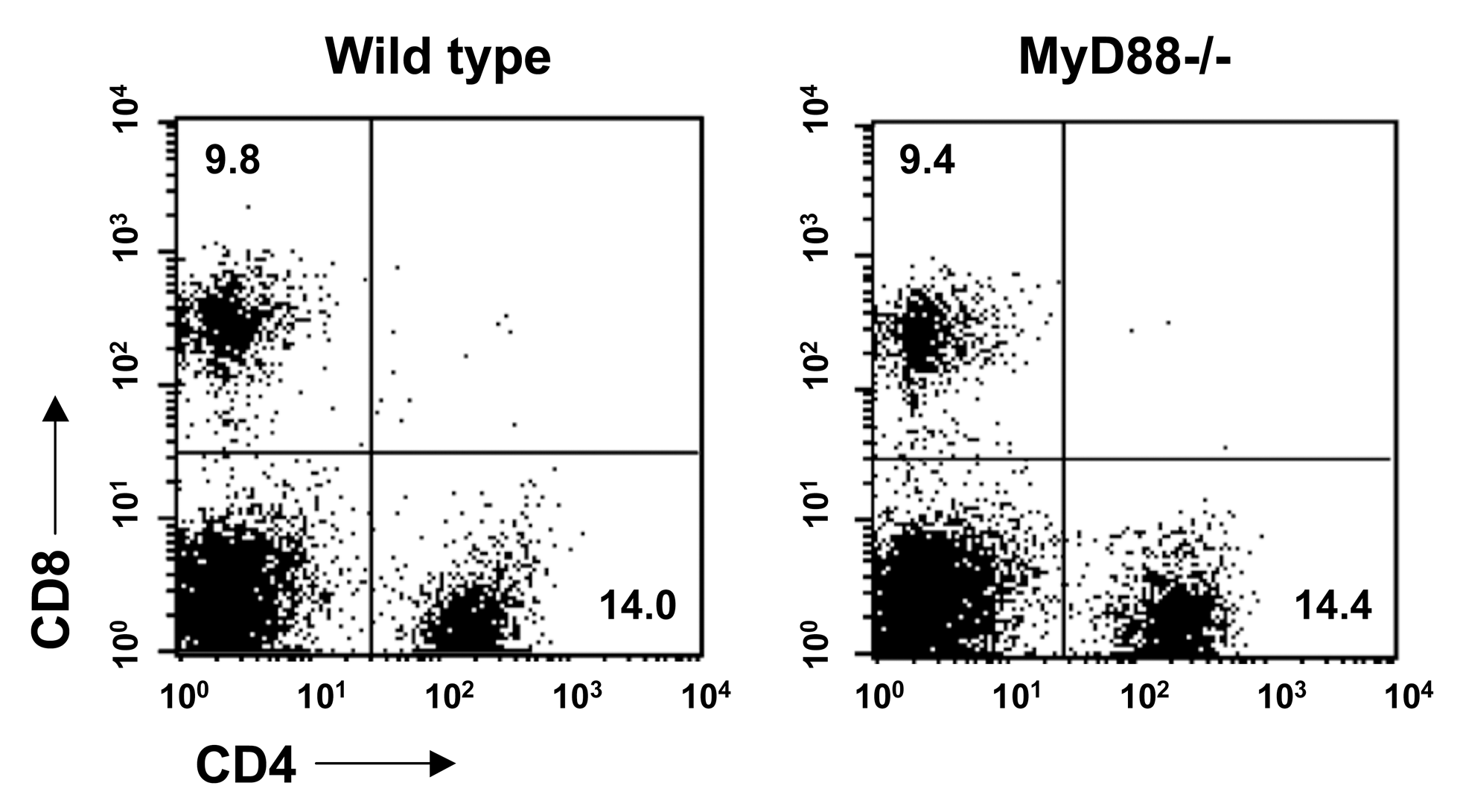

Supplement: Figure S2 — Similar CD4/CD8 T cell ratio between wild-type and MyD88-deficient mice during E. muris infection. Wild-type and MyD88-deficient mice were infected with E. muris for 10 days. Total splenocytes were analyzed by flow cytometry for surface expression of CD4 and CD8. Numbers in quadrants indicate the percentages of CD8+ and CD4+ cells among total splenocytes. Data are representative of at least three independent experiments. (0.45 MB TIF) [file pone.0011758.s002.tif]

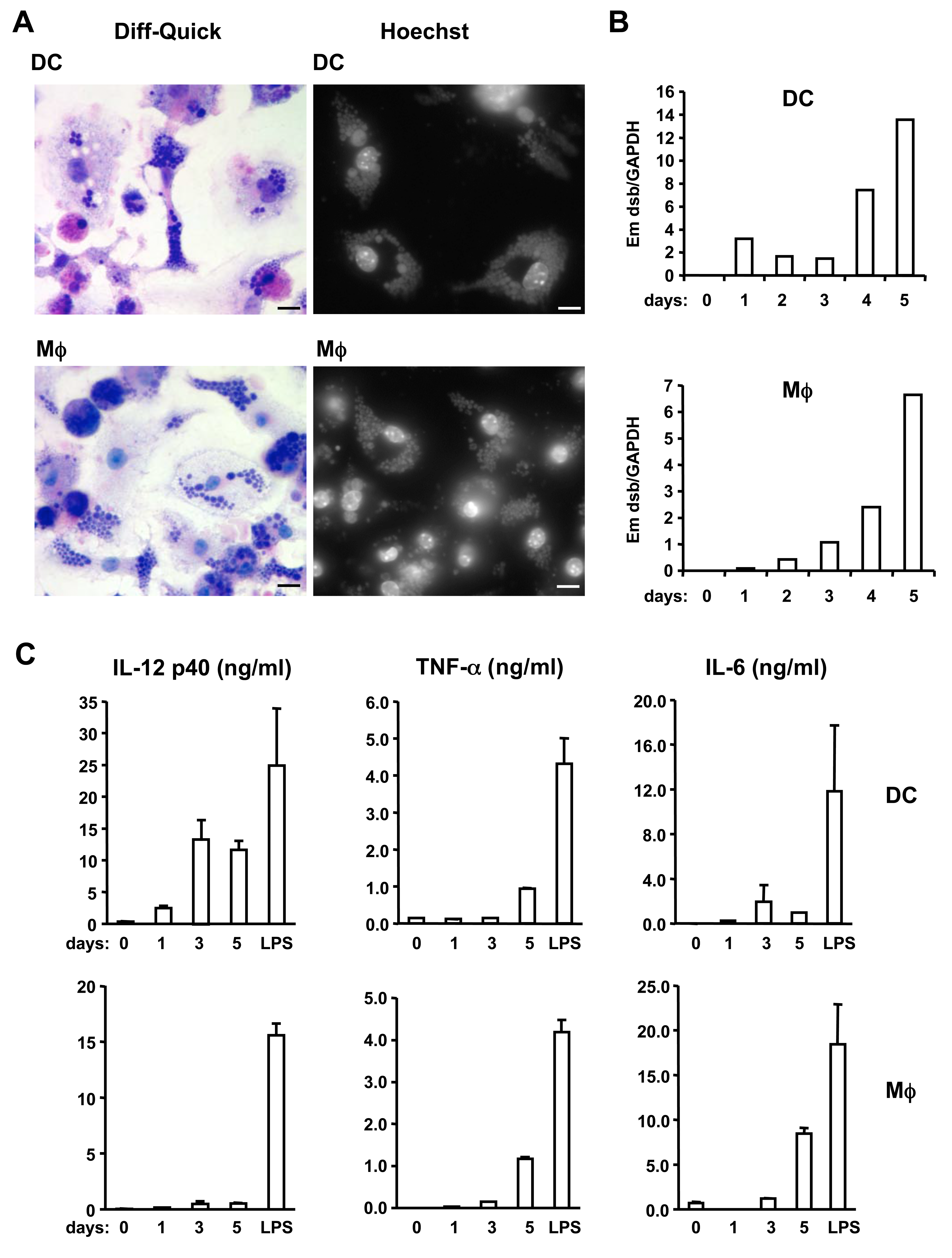

Supplement: Figure S3 — DCs but not macrophages produce IL-12 p40 efficiently during E. muris infection. Bone marrow-derived DCs and macrophages were infected with E. muris for the indicated time period. (A) Infected cells (5 days postinfection) were stained by Diff-Quick and Hoechst 33342 staining. Scale bar, 10 µm. (B) Bacterial loads in infected cells were quantified by qPCR using primers specific for the E. muris dsb gene. Data were normalized by qPCR data for the GAPDH gene in host genomic DNA. (C) Cytokine production from infected cells was assessed by ELISA. Error bars represent SD of triplicate samples. LPS (10 ng/ml) stimulation was used as a control. Data are representative of three independent experiments in A-C. (8.11 MB TIF) [file pone.0011758.s003.tif]

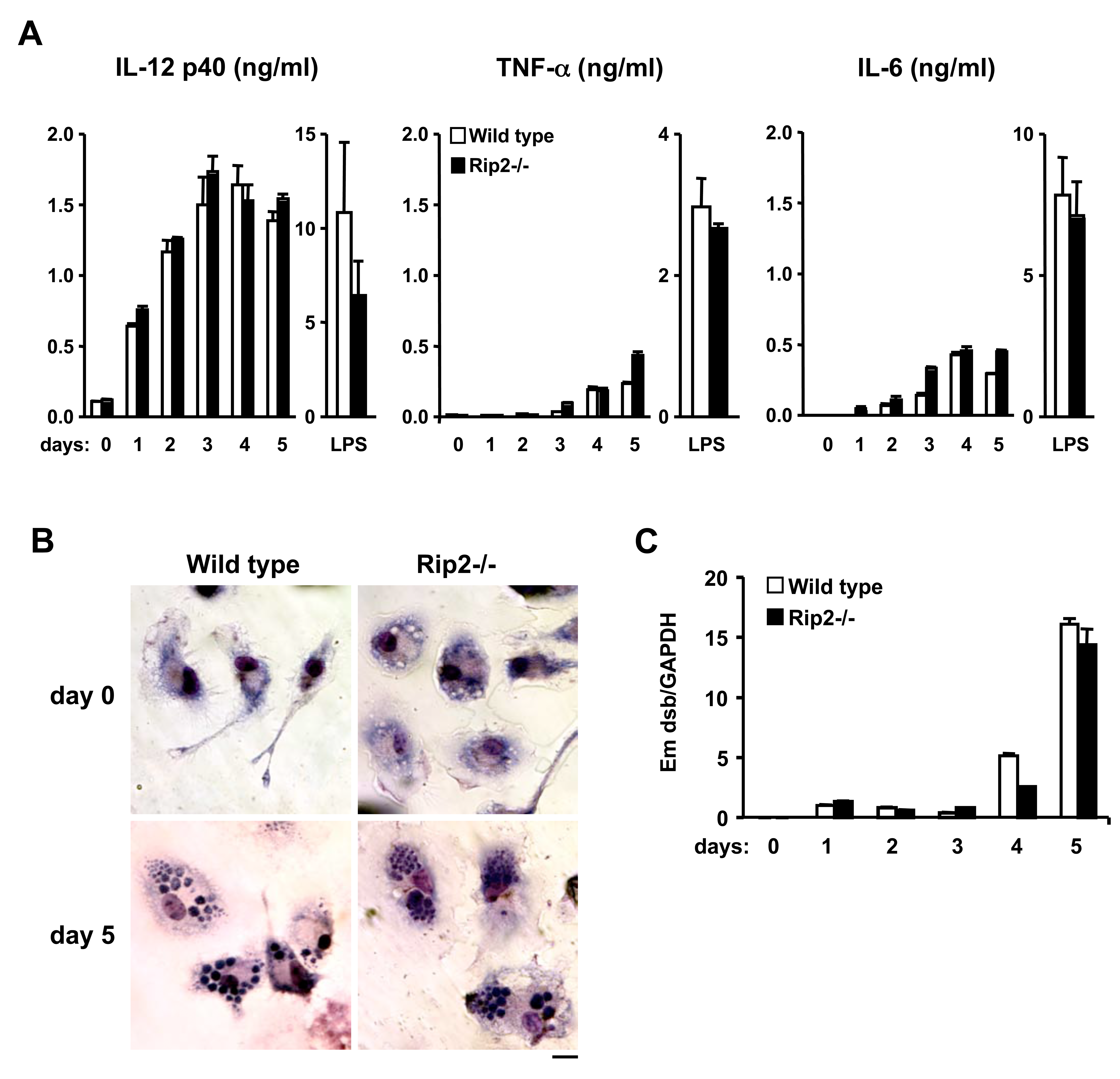

Supplement: Figure S4 — Rip2-independent E. muris recognition in DCs. Bone marrow-derived DCs from wild-type and Rip2-deficient mice were infected with E. muris for the indicated time period. (A) Cytokine production from infected cells was assessed by ELISA. LPS (10 ng/ml) stimulation was used as a control. (B) Infected cells (5 days post infection) were stained by Diff-Quick staining. Scale bar, 10 µm. (C) Bacterial loads in infected cells were quantified by qPCR using primers specific for the E. muris dsb gene. Data were normalized by qPCR data for the GAPDH gene in host genomic DNA. Error bars represent SD of triplicate samples. Data are representative of three independent experiments in A-C. (8.29 MB TIF) [file pone.0011758.s004.tif]

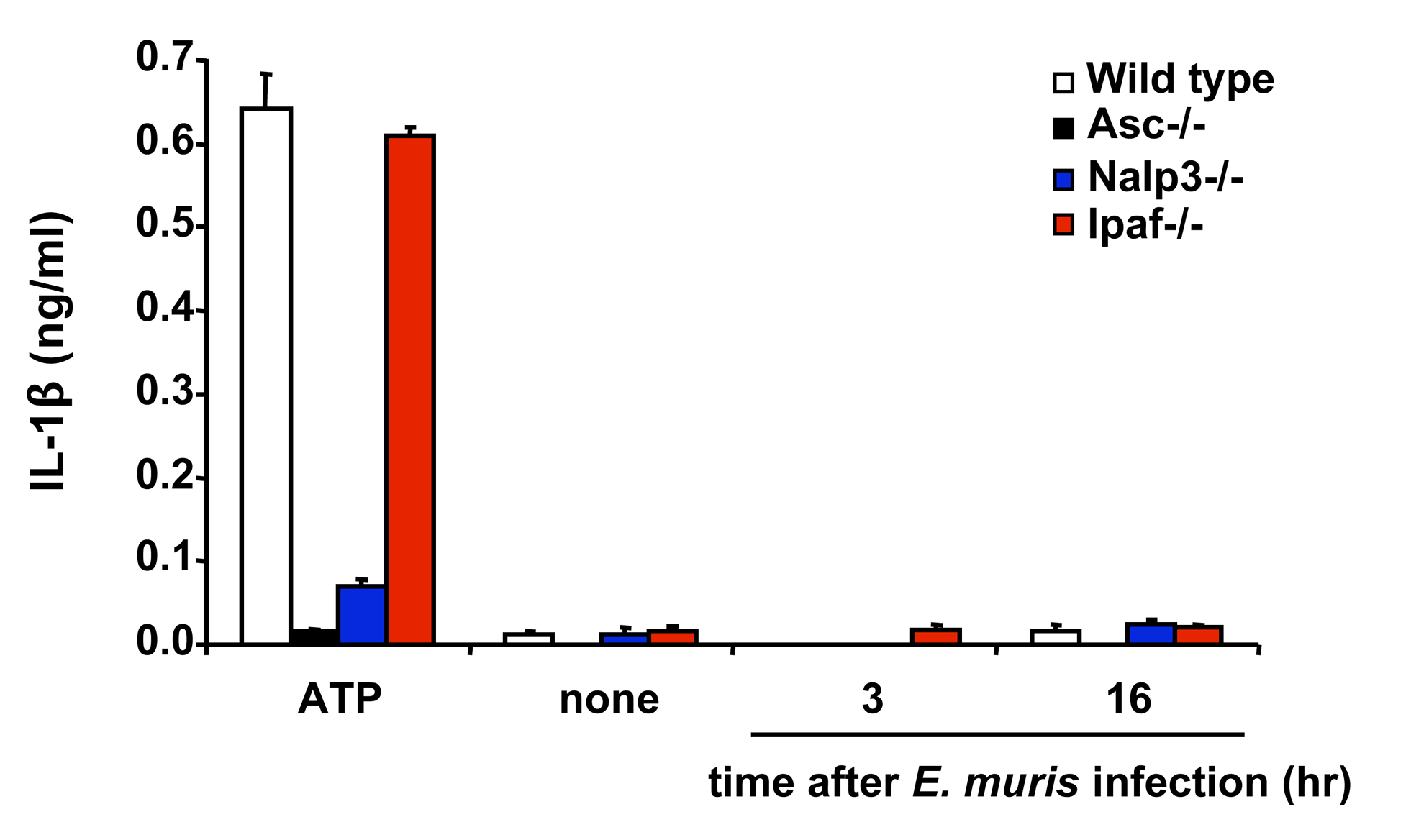

Supplement: Figure S5 — IL-1β production by Asc-, Nalp3-, or Ipaf-deficient macrophages infected with E. muris. Bone marrow-derived macrophages from both wild-type and Asc-, Nalp3- or Ipaf-deficient mice were treated with LPS (10 ng/ml) for 16 hours and then infected with E. muris in vitro for the indicated time period or stimulated with 2.5 mM ATP for 3 hours. IL-1β secretion in the culture supernatants was assessed by ELISA. Error bars represent SD of triplicate samples. Data are representative of three independent experiments. (0.18 MB TIF) [file pone.0011758.s005.tif]

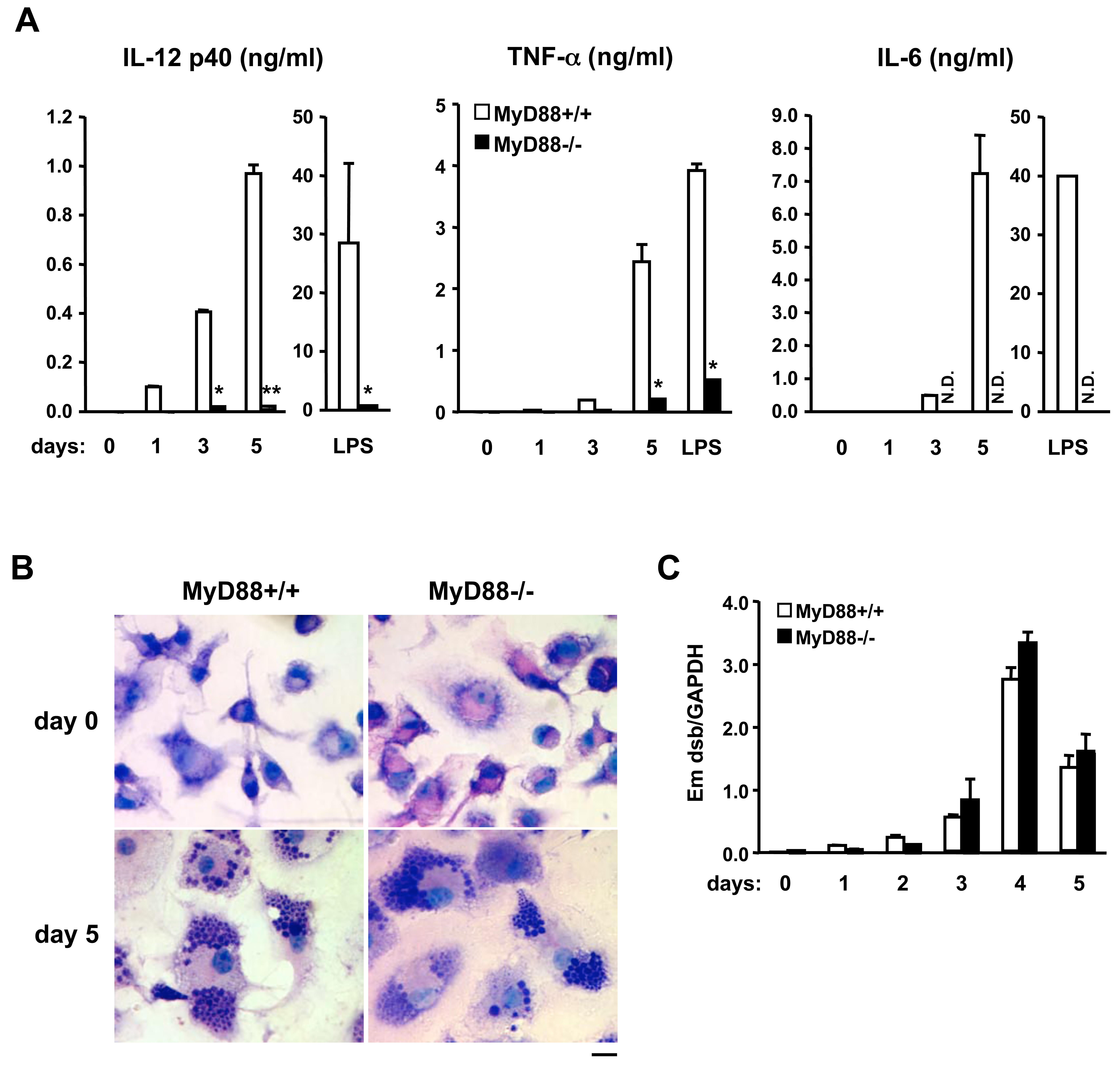

Supplement: Figure S6 — MyD88-dependent E. muris recognition in macrophages. Bone marrow-derived macrophages from wild-type and MyD88-deficient mice were infected with E. muris in vitro for the indicated time period. (A) Cytokine production from infected cells was assessed by ELISA. LPS (10 ng/ml) stimulation was used as a control. (B) Infected cells (5 days postinfection) were stained by Diff-Quick staining. Scale bar, 10 µm. (C) Bacterial loads in infected cells were quantified by qPCR using primers specific for the E. muris dsb gene. Data were normalized by qPCR data for the GAPDH gene in host genomic DNA. Error bars represent SD of triplicate samples. The p-values were determined by Student's t-test (MyD88+/+ vs. MyD88-/-). *P<0.05. **P<0.01. Data are representative of at least three independent experiments in A-C. N.D., not detected. (9.43 MB TIF) [file pone.0011758.s006.tif]

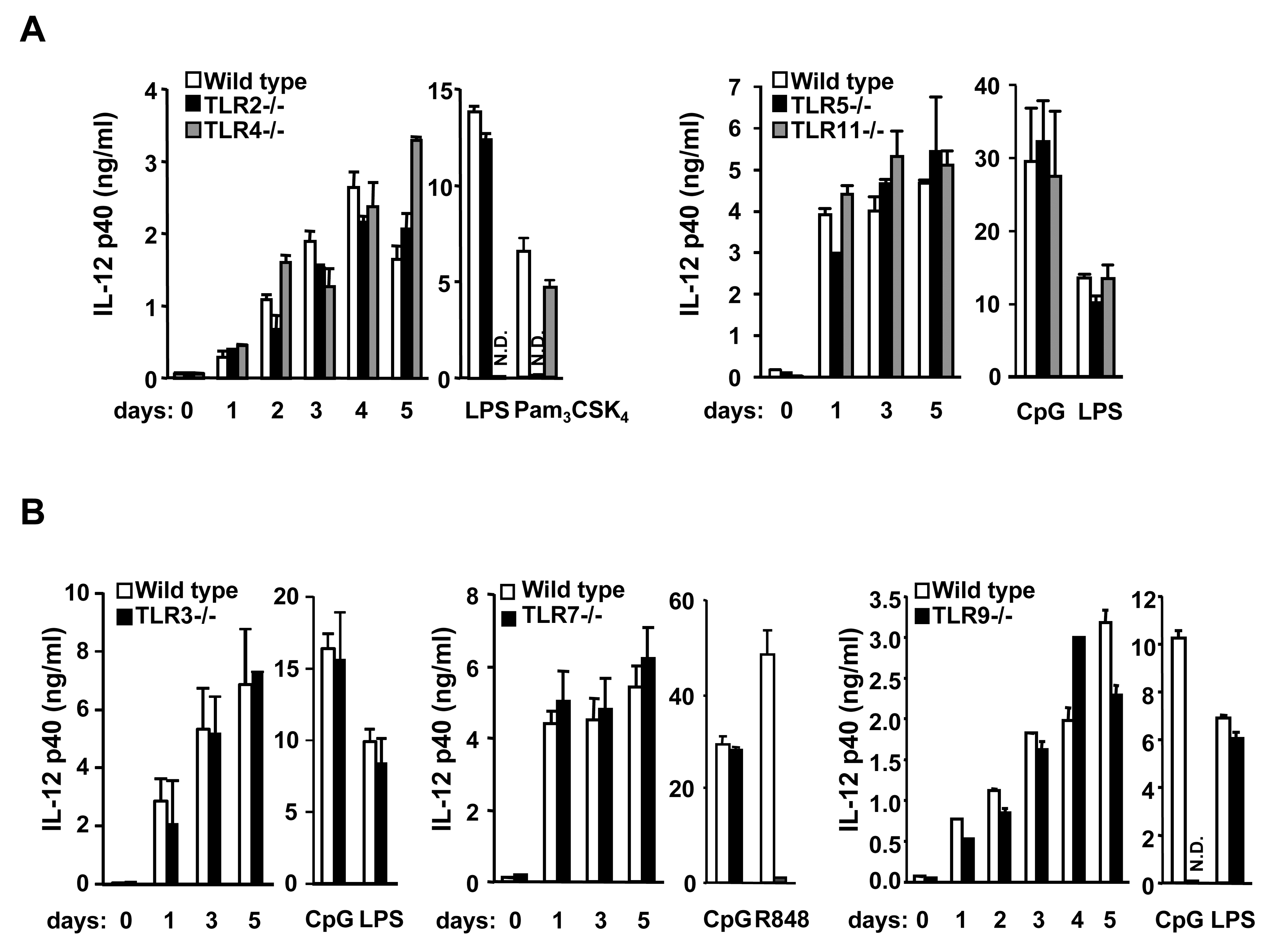

Supplement: Figure S7 — TLR2/3/4/5/7/9/11 do not play major roles in E. muris recognition. Bone marrow-derived DCs from wild-type, TLR2/4/5/11-deficient (A), or TLR3/7/9-deficient (B) mice were infected with E. muris for the indicated time period, and IL-12 p40 production from infected cells was assessed by ELISA. LPS (10 ng/ml), CpG (1 µM), R-848 (1 µM) and Pam3CSK4 (1 µg/ml) stimulations were used as controls. Error bars represent SD of triplicate samples. Data are representative of three independent experiments in A and B. N.D., not detected. (1.00 MB TIF) [file pone.0011758.s007.tif]
